# Experiences of Wives of Veterans with Post-Traumatic Stress Disorder: A Qualitative Study

**DOI:** 10.4314/ejhs.v33i2.19

**Published:** 2023-03

**Authors:** Davood Oudi, Seyyed Abolfazl Vagharseyyedin, Maryam Nakhaei, Aliakbar Esmaeili, Saeid Mohtasham

**Affiliations:** 1 School of Nursing and Midwifery, Birjand University of Medical Sciences, Birjand, Iran; 2 Department of Nursing, School of Nursing and Midwifery, Birjand University of Medical Sciences, Birjand, Iran; 3 Department of Psychiatry, School of Medicine, Medical Toxicology and Drug Abuse Research Center, Birjand University of Medical Sciences, Birjand, Iran; 4 Department of Psychiatry, School of Medicine, Birjand University of Medical Sciences, Birjand, Iran

**Keywords:** Stress Disorders, Post-Traumatic, Spouses, Veterans, Qualitative Research, Men

## Abstract

**Background:**

Living with post-traumatic stress disorder (PTSD) veterans imposes severe stress on relatives especially their wives. Despite of these difficulties these stratum of women suffer insufficient support and less attention. This study aimed to explore the experiences of wives of veterans with PTSD.

**Methods:**

This study was conducted in 2021 using qualitative content analysis approach. Participants were selected purposely from PTSD veterans' wives. Data were collected using semi-structured interviews with 18 wives of veteran men with PTSD. All the interviews were transcribed verbatim. Data were collected using in-depth and semi-structured interviews, up to the point of data saturation, and were analyzed by Graneheim and Lundman's content analysis method with the support of MAXQDA software.

**Findings:**

Through analysis of transcribed interviews, one theme and 4 categories and 13 sub categories were emerged : The theme called “abandoned in miserable life stream” and categories consist of “living in the cage of grief and regret”; “insufficient support resources”; “marital burnout”; and “self-sacrifice to maintain family cohesion”.

**Conclusion:**

The results of this study provide a broad range of context-specific challenges faced by wives of veterans with PTSD. In confronting with full of tensions life and lack of support, wives of veteran with PTSD are neglected and need more attention and support. It is essential for healthcare providers to be aware of the complex psychological and social conditions of wives of veterans with PTSD.

## Introduction

When the war ends and the participants return home, many of the physical and psychological effects of experiencing traumatic and life-threatening events on the battlefield reveal themselves to the individual and his relatives. Post-traumatic stress disorder (PTSD) is one of the major problems of experiencing war, which based on the latest statistics, affects approximately 4%-17% of all war veterans (1,2) .The prevalence of such disorder among the survivors of the Iran-Iraq war is higher than the global average prevalence and is estimated to be about 27.8% (3, 4). In traditional societies, veterans' wives are expected to care for their PTSD husbands, and without a sense of consent such comprehensive care leads to a compassion-driven fatigue (5). living with a veteran suffers PTSD and simultaneously playing the roles of mother, wife, nurse with economic and financial concerns causes severe tension and pressures on the veteran's spouse in long-term (6, 7). Consequently, the veterans' spouse has a significantly lower health and well-being compared to other women and experiences many challenges in life with the veteran (8).

PTSD not only has many complications for the affected person, but also causes many problems and challenges for his relatives and those around him. These complications include the negative effect of this disorder on the interpersonal relationships of the veteran and his spouse (9), destruction of marital functions (10), adverse effect on the mental health and well-being of the veteran's spouse (11) and suffering mental illnesses and problems such as depression (12). In other words, a veteran suffering from PTSD can be likened to a stone falling into a puddle of water that generates waves and affects not only the infected person but also his relatives and family (13). The complications caused by such disorder to the veterans' relatives and family are to the extent that the term secondary trauma or secondary victim is also used to describe the effects of this disorder on the victim's relatives (14). Regarding all the complications caused by the veteran with PTSD to the relatives and especially the spouse of the injured person, the voice of the veterans' spouse is almost neglected in the midst of the complications of the veteran. Few studies were conducted on the experiences of veterans' spouse regarding the severe effects of living with PTSD veterans on their wives (15–16).

Considering the close relationship of these effects to the context and culture, it seems that a qualitative study can properly collect more comprehensive information. Such a study will contribute to better understand and explain this concept (17). By explaining the challenges faced by the wives of PTSD veterans, a more comprehensive view and knowledge can be obtained on the problems of this group of women, who have received less attention. Regarding the above mentioned issues, we designed this qualitative study with the aim of exploring the challenges of wives of veteran with PTSD.

## Methods

**Study design**: This qualitative study was performed by conventional content analysis approach. Qualitative content analysis is a widely used method for interpreting the content of textual data through a process of systematic classification, coding, and identification of patterns or themes (18–19).

**Participants**: Inclusion criteria included living as a wife under one roof with a PTSD war veteran. They were selected from the spouses of veterans with PTSD who had a formal veteran record at the Mashhad and Birjand's Martyrs and Sacrificers Foundation. Interviews were conducted in medical or service centers with the veterans' foundation of mentioned cities.

**Data collection**: Data were collected by in depth, semi-structured interviews. Data collection was conducted between December 2020 and February 2022. Participants were selected based on the purposeful sampling method to match the aim of the study as closely as possible. The interview sessions were selected upon agreement with participants. All interviews were carried out a place where privacy could be assured. Interviews were recorded by digital recorder. To facilitate the interviews, we used an interview guide which was developed based on available scientific knowledge, along with discussions with experts in the field. The interview was initiated with an open-ended primary question such as, “please describe your experiences of living with your veteran's husband” and “please explain your difficulties and challenges in your life”. During data collection and analysis, some interview questions were modified or added to generate more information on potential emerging themes. Probing questions were asked for further clarification (e.g. “What do you mean?”, “Will you elaborate further?”). Silent probes allowed participants to reflect on descriptions. The duration of the interviews ranged from 60 to 90 minutes. All the interviews were conducted in Persian by the first author, a doctoral student trained in healthcare qualitative research. Data collection and analysis were simultaneous and during this process the topic guide was modified to explore emerging areas of interest. Sampling was determined using data saturation principles, with continual sampling until new data collected did not provide any new insights or themes on the phenomenon being studied. After 18 interviews, no new information was obtained.

**Data analysis:** Interview transcripts were initially read and re-read to make note of key words and phrases before importing them into MAXQDA 10 data management software for open coding and qualitative content analysis. Data were analyzed in five steps based on method proposed by Lundman and Graneheim (18). In the first step the interviews were read through and listened to several times by the first author to gain a sense of the whole. In the second step meaning units related to the aim were identified. In the third step the meaning units were condensed and labeled and finally coded on the basis of their content. Based on the codes, sub-categories and categories were developed in the fourth step. In the fifth step the categories were carefully discussed until main categories could be identified. All of the research team members validated the findings by reviewing and agreeing with the themes.

**Rigor**: The criteria proposed by Lincoln and Cuba (19) were used for establishing trustworthiness of the study findings using member checking, integrating the data sources and method integration, endorsing the coding by the colleagues familiar with qualitative research, coding, classifying similar codes and categories, transcribing the interviews as soon as possible and peer debriefing. In addition, the researcher carefully registered the research documentations to allow an external reviewer to evaluate the study. The effort was to choose participants with maximum variety of the duration of marriage, economic, social and educational backgrounds as well as PTSD symptoms severity.

**Ethical considerations:** The study was carried out in accordance with the Declaration of Helsinki. Study was approved by the Ethics Committee of Birjand University of Medical Sciences with the ethical code No. IR.BUMS.REC.1399.453. At the beginning of the interview sessions, purpose of the study was explained to all of participants and written consent was taken from the participants. Participants were aware about recording of their voice. They were assured that they could leave the study at any point and their identities and personal information would be kept confidential by researchers' team.

## Results

The Consolidated Criteria for Reporting Qualitative Research (COREQ) checklist was used to report important aspects of this study. The participants of this study comprised 18 wives of veterans with PTSD caused by war in Iran in 1980. Participants aged 37–70 years, and their education level ranged from under-diploma to diploma and one Bachelor's degree. From data analysis, a theme called “***Abandoned in Miserable life stream***” and four categories with 13 sub-categories were emerged (Table 1).

**Table 1:** Main theme, categories and subcategory of data analysis

Theme	
*Abandoned in Miserable life stream*	

Categories	Sub-categories
**Living in the cage of grief and regret**	Shadow of remorse on life
	Life with grief
	Being in the cage of life responsibilities
	Living with reluctance
**Insufficient support resources**	Weakness of the role of veteran's spouse in the family
	Inadequate government support
**Marital burnout**	Reduced well-being of veteran's spouse
	Lack of a positive feedback from veteran despite hard effort
	Constant tension in the veteran-spouse relationship
	Spouses' psychological fatigue from continuing to live with the veteran
**Self-sacrifice to maintain family** **cohesion**	Excessive effort to establish communication in the family
	Continuing to live with the veteran
	Patience to maintain family cohesion

**Abandoned in miserable life stream**: The main theme emerged in this study was abandoned in miserable life stream, which resulted from four categories: living in the cage of grief and regret, insufficient support resources, marital burnout and self-sacrifice to maintain family cohesion. The wives of PTSD veterans attempted to bring comfort to others through self-sacrifice during the hardships of life, and in this process, without even being seen sacrificed themselves and their rights with insufficient support resources, and thought only to continue living for others, and in the end they suffered from burnout by endless efforts without tangible results for themselves. The wives of PTSD veterans had self-sacrifice despite struggling with feelings of remorse and grief and burnout in their married life, trying to maintain the foundation of the family to the extent possible with inadequate support resources. They seemed to be abandoned helpless in the flow of trying to overcome life's problems and maintaining their families stem.

**Living in the cage of grief and regret:** This category includes four sub-categories of the shadow of remorse on life, life with grief, being in the cage of life responsibilities and living with reluctance. Based on the experiences of the participants, the veterans' wives were generally dissatisfied with the marriage with the veteran with PTSD and stated that if they had been aware of the problems of this marriage before marrying the veteran, they would not have gone through it, however, they currently had no choice but to continue living with the veteran, and considered quitting the veteran as falling from the frying pan into the fire, and thus continued to live a miserable life with a feeling of grief and remorse. One of the participants stated that (p3): *“I feel sad whenever I remember, and if the time machine had rewound to that time, I would not have married again, I am living with him now, but if I could divorce him, my hardships would end. It would be great.”* Another veteran's wife described it this way (p9): *“I did this (married) myself and my mother-in-law said my son is a psychic and a veteran and so and so, but I didn't think much about him and his problems as a veteran, I was a child, I didn't know about all these difficulties after marrying him, but what can I do now?” I do, I regret it, but I have to cope with it.”*

Facing difficult situations and experiencing caring for loved ones with chronic and incurable diseases and situations where the problem or defect remains unresolved and left untreated cause the formation of the phenomenon of chronic grief in relatives of the affected person. Due to the chronic and permanent nature of PTSD, living with a veteran with this disorder can facilitate the formation of grief in their spouses.

Regarding life with grief, the wife of one of the veterans stated (p11): *“Lack of joy and laughter has dried our throats; our house is a house of grief... I tell my son that your father is a PTSD veteran, and you have to accept that this house belongs to grief and cry, not laughter and joy.”*

In addition, due to the decline in the function of patients with PTSD and the veteran's inability to perform social and family duties and responsibilities, all these responsibilities are assigned to his wife and in addition to responsibilities related to veteran care, concerns related to the children and also the financial worries put high pressure on the veteran's spouse and this constant and endless preoccupation on the veteran's spouse causes them severe grief and mental fatigue. One of the participants stated in this regard (p13): “*My sons are single and their pressure is on my shoulders and the responsibility of two young son is so difficult, it is too difficult, I don't know how to express it.”*

The above-mentioned pressures and the pressures of caring for a veteran suffering PTSD leads to spouses' dissatisfaction with life and obligatory and reluctant toleration of life. Because separation from the veteran and leaving him causes more problems and worries for them and their children, the spouses of veterans continue to live and endure life with a veteran with PTSD with reluctance and unwillingness. One of the veterans' spouses said in this regard (p7): “*If I remember the past and my memories, I feel hatred towards myself and I get upset why I lived and I wish I didn't continue and I wish someone could come and separate me from my husband ... Now I tolerate because of my children, because they are grown and I cannot let them go.”*

**Insufficient support resources**: Veterans with PTSD suffer from a severe decline in performance in major and important areas of life such as work, social and marital areas, etc., and fail to effectively play the role of spouse, and this weakness in playing the role of spouse, imposes all the functions belonging to the veteran to his wives, and in other words, the wives of veterans with this disorder are obliged to compensate for the defects of the veteran's performance in addition to their duties who do not receive sufficient support and cooperation from their spouse. In such tortuous and difficult path of life, they are forced to lead and advance life alone or without sufficient support. One of the veterans' spouses explained about the defect in the role of the veteran's spouse (p14) as follows: *“I took control of life from the beginning, and I followed all the issues of life, children, financial issues, etc., and I tried for them, and in many cases, my husband (veteran) was not even aware of the problems and issues.”*

In addition, from the perspective of spouses of PTSD veterans the government support provided to the families and spouses of such veterans has not been effective and sufficient and does not solve their problems. Commenting on the inadequacy of government support, one participant stated (p2): *“Government services for veteran are of no use to me and I do not go to the [martyr] Foundation because they did nothing for his case ... so why should I go to the foundation when they do not do anything for me and it is useless?”*

**Marital burnout**: Tolerating remarkable psychological, physical, social and family pressures on the one hand and failing to achieve the desired result by the veteran spouse in life and marital relations on the other lead to a feeling of exhaustion and desperation in the veterans' spouses and in many cases they feel disproportion between the efforts made and the result obtained, which in total leads to marital burnout in the veterans' spouses. The relentless efforts of the spouses of PTSD veterans and the endurance of various pressures in their lives have often led to the loss of their physical and mental health and well-being, and most of the spouses of the veterans complained and suffered from various physical and psychological problems. One participant said in this regard (p8): *“I lost my legs health due to a lot of effort and work and moving heavy loads, I can't do all this work and I don't sleep many nights until morning because of pain in my knees.”*

In many cases, despite the great efforts of the veterans' spouses to improve the relationship with the veterans, they are not very satisfied with the services and care provided by their spouses, and sometimes these efforts are not accompanied by a positive feedback from the veterans. As a result, most of the veterans' spouses suffer burnout. One of the participants stated in this respect (p9): *“My husband (veteran) misbehaves me and slanders me, I wish we had a piece of bread but we ate peacefully ... He never thanked me but rebuked me ... When he hit me I had no shelter and he said it was your fault that you were flogged and I don't remember a time when he was satisfied with me and treated me well and appreciated me.”*

Suffering with PTSD often has long-term, destructive effects on the mind and psyche of the sufferer. One of the most important adverse effects is aggression and loss of control over the impulse in the affected person, so that a nervous dispute and conflict with the spouse is a daily incidence in the life of the veteran family and this issue itself causes a lot of pressure on the veteran spouse and subsequently leads to burnout.” Another veteran's spouse said in this regard (p17): *“The war is still going on in our house and there is a war as long as we are ... I also told my son-in-law that if you come to this house, you will come to the battlefield and be careful ... “This family is still on the front lines of the war and there is no end to it and there is no freedom.”*

Excessive fatigue due to various responsibilities such as spouse's illness, maternal duties, spouse duties, financial concerns and other concerns have caused severe mental fatigue to the veterans' spouses and their subsequent burnout, as one of them said (p10): *“Sometimes I get tired and because I am one person in life, outside the house, in the hospital and everywhere, I am responsible and it has been the same since the beginning of the marriage and Mr. B (veteran) did not work for more than two or three years and was then disabled and the whole life burden fell on me and I got very tired, and I'm worried about my children's' future, and that's bothering me a lot.”*

**Self-sacrifice to maintain family cohesion**: Due to the negative consequences of PTSD on the veteran's relationship with relatives and friends and as a result of its destructive effect on family strength and cohesion, veterans' wives compensated efforts to maintain family cohesion and stability. Veterans' wives were forced to work beyond their power and forget their personal rights and self-sacrifice to maintain and establish communication in the family and therefore, maintain family cohesion. In this regard, one of the participants explained (p11): *“When there is a dispute or discussion, I talk to him and make it up to him and say I was guilty, I make it up to him even if he is guilty.”*

Considering the sacrifice of their rights and desires in life, veterans' wives considered others, especially their children and spouses, as their reason for continuing to live, and in many cases endured life in order to build their children's future and prevent damaging it and tried to preserve it. They devoted themselves to their children and veterans and continued to live and endure to maintain their health and provide services to them. One of the participants said (p6): *“Once my mother said: let's go to get your divorce tomorrow and I said no, I do not want to separate. He will get hurt ... Sometimes I feel pity for him and I tell myself that if I get separated from him, he will be taken to a sanatorium and no one will care him and I do not like to leave him alone, I stayed with him for himself (the veteran).”*

Patience in the veteran's misbehaviors and life problems was another effort made by the veterans' spouses to maintain the cohesion of life. They ignore their rights in life and thought only of patience to maintain family cohesion. One of them said (p13): *“Early in the marriage, he used to come home from work and beat me so much that my body turned black and my finger broke once, but I stayed silent and tolerated and did not answer.”*

## Discussion

This study aimed to explore the experiences of wives' challenges of living with a PTSD veteran. Despite of abundant pressures and difficulties, these stratums of women has not been noticed and have been oppressed and neglected and they don't receive a proper position commensurate with their efforts in our society, families and related organizations too. These women not only are neglected but also their efforts don't change their life and always suffer feelings remorse, grief and disappointment from the pass they have chosen for their life. A study findings revealed severe mental pressure and insufficient social support can lead to depression and spiritual pain in PTSD veteran's spouses (20). Another study results showed meaningful increase of mood disorders and depression scores mean in spouses of PTSD veterans compared with spouses of none PTSD veterans (21). Earlier studies explain abundant war complications on wives of PTSD veterans as permanent and chronic disappointment, feeling defeated, chronic sorrow and remorse and anger, while there was lack of knowledge and consideration about war complications on women mentioned above. In other word this stratum of ladies missed and abandoned by society and politicians too (22, 23). In line with the results of present study, it has been previously shown that living with combat PTSD patients had negative effects on the mental condition of the family members, especially their wives, and they suffered from psycho-social problems (24).

Participating in the war to protect the homeland has made the veterans respect and have a special respect in the culture and society.. Veterans receive government and charities supports and benefits, but their wives are neglected and don't receive these supports and benefits while they always accompanies veterans especially in our culture. In religious and traditional countries, veterans' wives have extra responsibilities in their life added to their marital and maternal ones and this lead to abundant pressure on them (5). In other word, despite of great responsibilities and complications of veteran's wives, these women do not receive enough support from the society, veteran and government agencies and suffer from loneliness, unsafe and abandonment feelings permanently (5, 20). Function impairment in PTSD veterans imposes parental, marital, caring and financial duties on their neglected spouses (15). Veteran's wives do not receive enough support and services despite of their multiple duties and complications in their life (8). Great pressure and stress on veteran's wives cause adverse effects as decreasing quality of life (16), poor mental health and marital burnout in these women (12, 25).

In religious and traditional cultures these stratum of women's out of power efforts to manage great life challenges and also their multiple roles and duties along with disproportionate and improper feedback from husband and society cause marital burnout and spiritual fatigue in them (5). Wives of veteran with PTSD, in most cases suffer negative feelings such as blaming, guilt (26, 27), contradiction, anger, lack of intimacy and close relation and as a whole their life is challenging and stressful (28). Living with veteran can cause poor control on time and environment and turning away relatives from personal wishes, desires and space and poor welfare and comfort as a result (29, 30). Despite the great efforts and sacrifices of veteran's wives in marital life to maintain family cohesion and stability, they don't receive proper and worthy attention from their relatives and society too .in attention to researches results mentioned above we can say veterans wives are neglected and abandoned war victims who are not seen and supported properly (1). A number of limitations in the present study deserve to be mentioned. A limitation of current study may be recall bias since participants addressed their current experience and sometimes previous experiences also. Also, the results of this study might not be generalizable to all spouses of veteran men with PTSD in other countries that incorporate different ethnicity, religions, cultures, economic, and political backgrounds, which is another limitation of this study.

In conclusion the results of this study provide a broad range of context-specific challenges faced by wives of veterans with PTSD. According to the results of present study it seems that wives of veteran with PTSD, as they are closest persons to veteran, are the most affected ones by war after veterans, in other word they are second war victims. This stratum of women have been confronted with great pressures and challenges in marital life with PTSD veterans and despite of these challenges and burnout they don't give up and defense from their family coherence by self-scarifying as a solder without sufficient support. It is essential for healthcare providers to be aware of the complex psychological and social conditions of wives of veterans with PTSD.
